# Tuning the Magnetic Properties of Cr_2_TiC_2_T_x_ through Surface Terminations: A Theoretical Study

**DOI:** 10.3390/nano12244364

**Published:** 2022-12-07

**Authors:** Shaozheng Zhang, Yuanting Zhou, Xing Liang, Yulin Wang, Tong Wang, Jianhui Yang, Liang Lv

**Affiliations:** 1 College of Teacher Education, Quzhou University, Quzhou 324000, China; 2 College of Chemical and Material Engineering, Quzhou University, Quzhou 324000, China; 3 Ningbo Institute of Materials Technology and Engineering, Chinese Academy of Sciences, Ningbo 315201, China

**Keywords:** density functional theory, two-dimensional magnetic materials, MXene, spin device, surface termination

## Abstract

Recently, magnetic two-dimensional Cr_2_TiC_2_T_x_ MXenes with promising applications in spin electronics have been experimentally confirmed. However, the underlying magnetic mechanism needs to be further investigated. Along these lines, in this work, the magnetic properties of Cr_2_TiC_2_O_n/4_F_2−n/4_ and Cr_2_TiC_2_O_n/4_ structures were simulated through first-principle calculations using the GGA+U approach. The values of 4.1 and 3.1 eV were calculated for the Hubbard U of Cr and Ti, respectively, by applying the linear response method. Interestingly, the Cr_2_TiC_2_O_n/4_F_2−n/4_-based configurations with low O content (*n* ≤ 4) exhibit antiferromagnetic behavior, while the majority of the respective configurations with high O content (*n* ≥ 5) are ferromagnetic. As far as the Cr_2_TiC_2_O_5/4_F_3/4_ structure (*n* = 5) is concerned, the value of about 2.64 μ_B_ was estimated for the magnetic moment of the Cr atom. On top of that, the Curie temperature lies within the range of 10~47 K. The extracted theoretical results are in good agreement with experimental outcomes of the Cr_2_TiC_2_O_1.3_F_0.8_-based structure. From the simulated results, it can be also argued that the magnetic moment of Cr atoms and the Neel temperature can be directly tuned by the active content of O atoms. The conductivity of both Cr_2_TiC_2_O_n/4_F_2−n/4_ and Cr_2_TiC_2_O_n/4_ configurations can be regulated by the externally applied magnetic field, while the density of states around the Fermi level shifted significantly between ferromagnetic and antiferromagnetic arrangements. The acquired results provide important theoretical insights to tuning the magnetic properties of Cr_2_TiC_2_T_x_-based structures through surface termination mechanisms, which are quite significant for their potential applications in spin electronics.

## 1. Introduction

Since the discovery of two-dimensional early transition metal carbide materials named MXene by Gogotsi and coworkers [1,2], extensive application perspectives in many fields including energy storage [3], catalysis [4], sensors, and electronics have emerged [5], which have revealed the advantages of their unique properties [6,7]. The theoretical and experimental demonstrations of magnetic MXene properties promote their application in the field of spin electronics [8,9,10,11,12,13,14]. For example, the existence of half-metal characteristics renders the double-layered Cr_2_NO_2_ structure an ideal ultrathin spin-filtering material accompanied by low magnetic turnover energy, high switching ratio, and low energy consumption [15].

Consequently, investigating the magnetic properties of MXene is considered of utmost importance to further promoting its application perspectives. Thus, tremendous efforts have been devoted to exploring the magnetic properties of Cr-based MXenes for exploiting their potential integration in spin electronic devices. For instance, when surface functionalization groups of F, OH, H, or Cl are employed for the Cr_2_C MXene structure, antiferromagnetic properties can be attained, while various magnetic and electronic properties such as the energy gap are tunable by changing the type of the functional terminations and dopants [16,17,18]. The variations of the Cr 3d orbitals, which are induced by the surface terminations, are regarded as the main factor for the abovementioned transformations of the electronic and magnetic properties. By performing first-principle simulations, it has been also predicted that the enforcement of an external strain smaller than 5% can induce ferromagnetic to antiferromagnetic transition in Cr-based MXene structures [5,9]. For Cr and Mn-based MXenes, the strength of the spin–orbit interaction can be manipulated through changing surface terminations, and sufficient anisotropy with long-range magnetic order can be obtained [12]. For example, both Cr_2_NO_2_ and Mn_2_NO_2_ configurations are ferromagnetic with magnetic anisotropy energies higher than 63 μeV per Cr atom [12].

Theoretical reports in the literature have shown that the excellent magnetic properties of Cr-based MXene can be further optimized through Janus terminations [19,20]. For instance, the asymmetric surface terminations of the Janus Cr_2_C system can improve magnetic anisotropy and hence impose a more stable 2D magnetic ordering [20]. Remarkably, the value of about 400 K was found for the Neel temperature of various Janus MXene configurations such as Cr_2_CFCl, Cr_2_CClBr, Cr_2_CHCl, Cr_2_CHF, and Cr_2_CFOH by He and coworkers [21]. Additionally, doping with electrons or holes has been proven through the first-principles method as an effective strategy for controlling the spin carrier’s orientation properties [21]. The magnetism can be controlled via superconductors [22]. Moreover, by carrying out density functional theory calculations, He and coworkers also demonstrated that the Cr_2_TiC_2_FCl structure is a kind of novel bipolar antiferromagnetic semiconductor since its gate voltage can be tuned using the spin orientation technique [19]. From the abovementioned studies, it is apparent that the magnetic properties can be directly tuned, which is of vital importance to the potential incorporation of 2D high-temperature spin-polarized materials within next-generation spintronic devices [21,23].

The magnetic properties of the Cr_2_TiC_2_T_x_-based structure have been also experimentally demonstrated by Gogotsi and coworkers. More specifically, the authors found that the Cr_2_TiC_2_T_x_ configuration is a kind of spin-glass with a magnetic transition temperature of 30 K [11]. On top of that, by performing in situ probe analysis, it was revealed that the O and F terminations on the surface of the Cr_2_TiC_2_T_x_ structure were about 62% and 38%, respectively. F atoms can be desorbed at 600 ℃, while O atoms remain relatively stable at 700 ℃ [14].

Under this direction, in this work, the underlying mechanism of the enhanced magnetic properties of Cr_2_TiC_2_T_x_ was thoroughly analyzed by using the density functional theory method. Then, the impact of O terminations on both electronic and magnetic properties was analyzed to tune the Neel and Curie temperatures of the Cr_2_TiC_2_O_n/4_F_2−n/4_ structure through surface terminations.

## 2. Calculation Methods and Models

Density functional theory calculations were performed by employing projector-augmented wave (PAW) potentials for all calculations [24], which was realized through the Vienna ab initio simulation package (VASP) [25,26,27]. The kinetic energy cutoff was set to a value of 500 eV. The ionic relaxation process was performed until the force on each atom was less than the value of 0.02 eV/Å. The k-points meshes were chosen by using the Monkhorst–Pack method [28], while the k-point grid spacing in each direction of the reciprocal space was smaller than 0.03 Å^−1^. A vacuum layer thicker than 15 Å was also assumed along the *z*-axis to minimize the interactions between the two nearest layers. The exchange–correlation energy was handled with the generalized gradient approximation (GGA-PBE) functional [29]. The GGA + U approach was also used for Cr and Ti 3d electrons [30], and the U value was calculated by employing the linear response approach [31]. Charge and magnetic moment on Cr ions are obtained from the projection of the occupied wavefunctions onto spherical harmonics that are non-zero within spheres of a radius centered at a Cr ion, which can be obtained from OUTCAR file of VASP.

**Figure 1:**
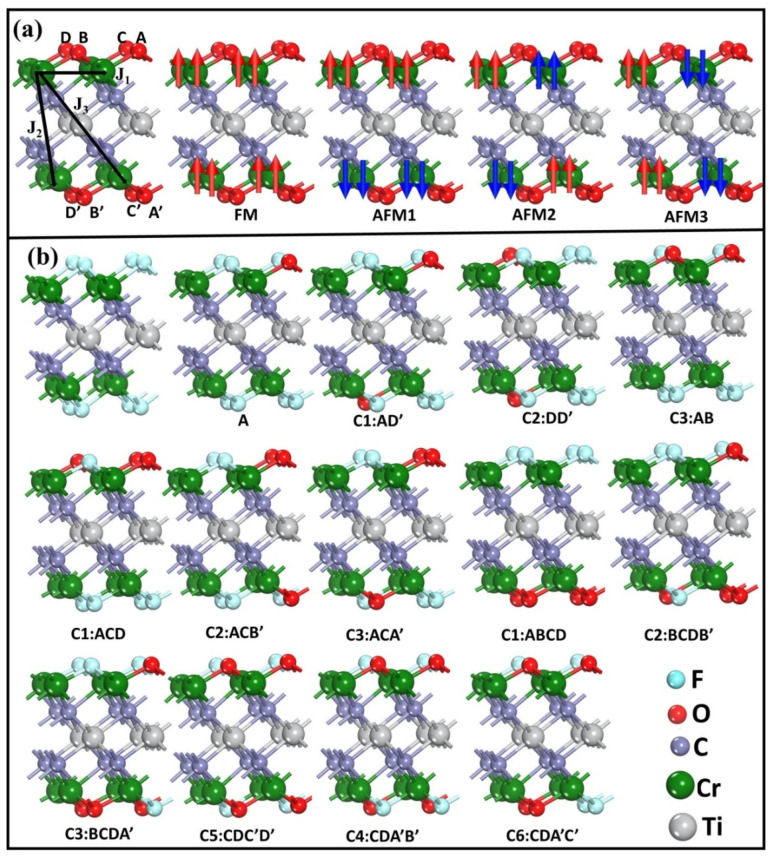
Side views of (**a**) the magnetic arrangements and adsorption sites, and (**b**) configurations of Cr_2_TiC_2_O_n/4_F_2−n/4_ (*n* = 0, 1, 2, 3, 4).

In this work, the surface terminations of both O and F ions were assumed to take place in hollow sites, which is in direct agreement with former theoretical results [10,32]. Moreover, 2 × 2 supercells with the following chemical formula were considered: Cr_2_TiC_2_O_n/4_F_2−n/4_ (*n* = 0, 1, 2, 3, 4, 5, 6, 7, and 8). According to former research [10], hollow site A, below which is a Ti atom, is energetically preferable for the adsorption F or O atoms. Here, three different adsorption models of Cr_2_TiC_2_O_2_ and Cr_2_TiC_2_F_2_ were tested. O and F atoms energetically prefer to be in the hollow site A as shown in Figure S1. As can be ascertained from Figure 1a, each side possesses four adsorption sites, whereas all the possible adsorption configurations of O and F ions were considered. Figure 1b depicts the atomic configurations of the Cr_2_TiC_2_O_n/4_F_2−n/4_ (*n* = 1, 2, 3, and 4) configurations. For the values of *n* = 5, 6, and 7, the configurations are the same as the case of *n* = 3, 2, 1, while O and F ions interchange positions. Moreover, ferromagnetic (FM) and three different antiferromagnetic arrangements (AFM1, AFM2, and AFM3) were considered in this work, as is shown in Figure 1a. The lattice parameters were fully optimized for different Cr_2_TiC_2_O_n/4_F_2−n/4_ configurations.

## 3. Results and Discussion

### 3.1. The Hubbard U of Cr and Ti Atoms in Cr_2_TiC_2_T_x_ Systems

To obtain theoretical results consistent with the respective experimental values, it is quite significant to find a suitable Hubbard U in the GGA+U approach. By enforcing the linear response approach developed by Cococcioni and Gironcoli [31], the following values of Hubbard U for the Cr (U_Cr_) of Cr_2_TiC_2_O_2_, Cr_2_TiC_2_F_2_, Cr_2_TiC_2_(OH)_2_ systems were calculated as 4.2, 4.0, 4.0 eV, respectively, as can be observed from Figure S2. If the surrounding chemical bonds were inconsistent, small differences in the localization of Cr 3d electrons among the Cr_2_TiC_2_O_2_, Cr_2_TiC_2_F_2_, Cr_2_TiC_2_(OH)_2_ systems were found, which led to the variation of Hubbard U. However, such weak variations have negligible impact on both the magnetic and electronic properties. The same trend also exists in the Hubbard U for Ti atoms, while U_Ti_ is smaller than U_Cr_. Moreover, small variations of U_Cr_ around 4 eV do not cause significant variation of magnetic moments or magnetic phase transition between FM and AFM, as shown in Figure S3. For Cr_2_TiC_2_F_2_, the energy of AFM1 was lower than that of FM states as U_Cr_ varied from 2 to 6 eV. Thus, in the latter calculations, the Hubbard U values for Cr (U_Cr_) and Ti (U_Ti_) atoms were set as 4.1 and 3.1 eV, respectively.

### 3.2. The Magnetic Properties of Cr_2_TiC_2_O_n/4_F_2−n/4_ and Cr_2_TiC_2_O_n/4_

**Figure 2:**
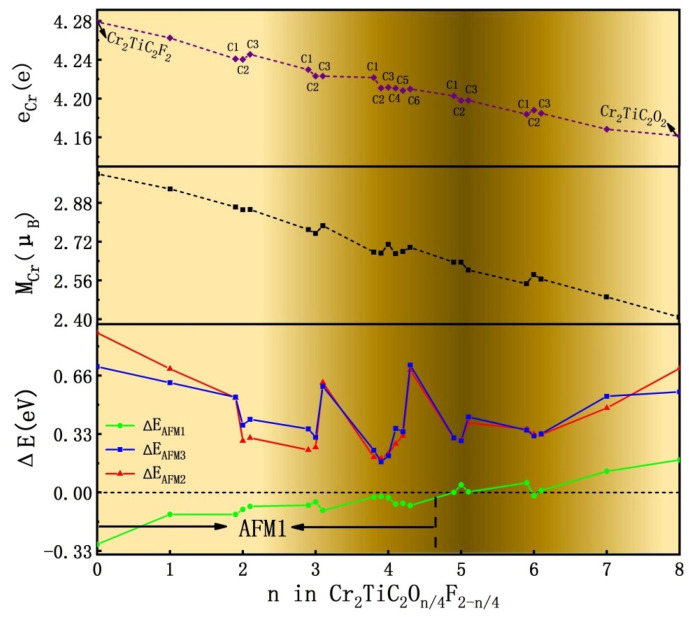
(**Top panel**) Distribution of the number of electrons of Cr atoms (e_Cr_). (**Middle panel**) Distribution of the magnetic moment of Cr atoms (M_Cr_). (**Down panel**) Energy difference of AFM1 (ΔE_AFM1_), AFM2 (ΔE_AFM1_), AFM3 (ΔE_AFM1_) with FM state of the Cr_2_TiC_2_O_n/4_F_2−n/4_ system.

The magnetic properties of Cr_2_TiC_2_F_2_ and Cr_2_TiC_2_O_2_ with *n* = 0 and 8 for Cr_2_TiC_2_O_n/4_F_2−n/4_ systems were calculated first. The ground state of the Cr_2_TiC_2_F_2_ is AFM1 with one Cr atomic layer in spin-up and another Cr atomic layer in spin-down state. This outcome is in direct agreement with former theoretical results [10,32], while the ground state of the Cr_2_TiC_2_O_2_ structure is FM. This result indicates that an AFM-FM phase transition takes place as *n* is increased from the value of 0 to 8. In detail, AFM1 is energetically favorable for the Cr_2_TiC_2_O_n/4_F_2−n/4_ configuration when *n* ≤ 4, whereas FM is energetically favorable for most Cr_2_TiC_2_O_n/4_F_2−n/4_ systems when *n* ≥ 5, as is shown in the down panel of Figure 2. In general, the energy difference between FM and AFM1 arrangements (ΔE_AFM1_) is increased by employing *n* values from 0 to 8, and is close to zero when *n* = 4, 5, and 6. From the reported experimental results in the literature it has been revealed that the surface terminations of the Cr_2_TiC_2_T_x_ structure are F and O atoms with the following initial composition: Cr_2_TiC_2_O_1.3_F_0.8_ [11,14]. This composition corresponds to the value of *n* = 5 for the Cr_2_TiC_2_O_n/4_F_2−n/4_ system. In fact, since the growth of the material is significantly affected by various environmental factors such as temperature, its surface terminations do not present a uniform distribution. Thus, the respective chemical compositions of the Cr_2_TiC_2_O_n/4_F_2−n/4_ system with *n* = 4, 5, and 6 are all most likely to exist in the reported experimental results. Furthermore, the Cr_2_TiC_2_O_n/4_F_2−n/4_ structure prefers to exhibit AFM1 and FM properties when *n* = 4 and 5, which induces a spin-glass state for the Cr_2_TiC_2_O_1.3_F_0.8_ structure.

The average magnetic moment of Cr atoms (M_Cr_) in the Cr_2_TiC_2_O_n/4_F_2−n/4_ systems is decreased as *n* becomes bigger, as is shown in Figure 2. This effect can be attributed to the existence of more unoccupied orbitals of O atoms than that of F. It is well-established that electrons are transferred from Cr atoms to O or F atoms when Cr-O or Cr-F bonds are formed. Thus, as the *n* value is increased, the number of Cr 3d electrons is decreased, which leads to the reduction of M_Cr_. More specifically, for the value *n* = 5, M_Cr_ is about 2.64 μ_B_, which is in good agreement with the reported experimental results of the Cr_2_TiC_2_O_1.3_F_0.8_ structure, which is 2.73 μ_B_ [11].

As far as the terminations of F atoms on the surface of the Cr_2_TiC_2_O_1.3_F_0.8_ structure are concerned, it can be desorbed at the temperature value of 600 °C. The magnetic properties of the Cr_2_TiC_2_O_n/4_ configuration with some uncovered hollow sites on its surface were also analyzed in this work. Similar to the Cr_2_TiC_2_O_n/4_F_2−n/4_ system, ΔE_AFM1_ is increased as *n* becomes bigger, as can be observed from Figure S4. Interestingly, the Cr_2_TiC_2_ structure prefers antiferromagnetic arrangement (AFM1). Different from the Cr_2_TiC_2_O_n/4_F_2−n/4_ system, the antiferromagnetic to ferromagnetic transition takes place at the value of *n* = 4 for the Cr_2_TiC_2_O_n/4_ systems, while the majority of the examined configurations prefer the ferromagnetic state when *n* ≥ 5. Generally, M_Cr_ is decreased as *n* is increased coupled with the decreasing pattern of e_Cr_.

**Figure 3:**
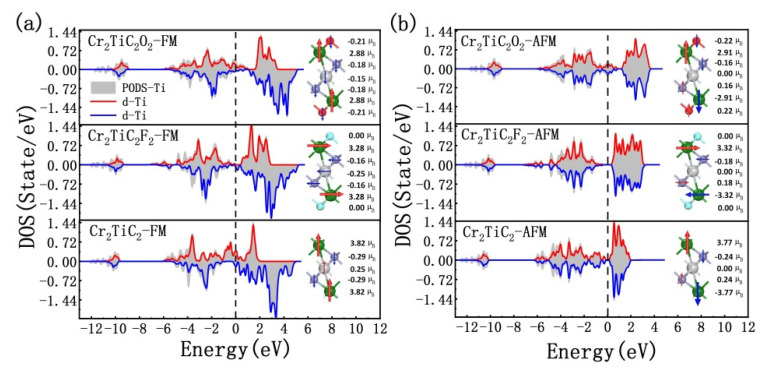
Projected density of states of Ti atoms and magnetic moments in the Cr_2_TiC_2_F_2_ and Cr_2_TiC_2_O_2_ structures with FM (**a**) and AFM1 (**b**) arrangements.

Due to the resonance between electron orbitals of Cr and nearby atoms, all nearby atoms except F around Cr atoms are magnetized weakly with the opposite magnetic moment to Cr atoms, as is shown in Figure 3. For the O and C atoms, the extracted magnetic moments are about 0.16~0.29 μ_B_. Although Ti atoms can also be magnetized with a magnetic moment among 0.15~0.29 μ_B_ in FM arrangements, the magnetic moment is zero in AFM1 arrangements. Such a characteristic can also be captured in the calculated density of states for Ti atoms. As is shown in Figure 3a, the spin-up and spin-down densities of states for Ti electrons in FM arrangements are asymmetrical, while symmetrical distributions were calculated for AFM1 arrangements, as is depicted in Figure 3b. Additionally, the super exchange interaction between Cr atoms in the up and down layers may affect the magnetic arrangements of both Cr_2_TiC_2_O_2_ and Cr_2_TiC_2_F_2_ systems [33]. According to Anderson’s theoretical analysis, oxides prefer the FM arrangement when the number of electrons of the magnetic atoms is less than the half-filled shells, whereas the antiferromagnetic arrangement is preferred when the number of electrons of the magnetic atoms is more than the half-filled shells [33]. As far as the Cr_2_TiC_2_O_2_ and Cr_2_TiC_2_F_2_ systems are concerned, the number of electrons of the Cr atoms is close to the half-filled shells. Cr atoms in the Cr_2_TiC_2_F_2_ system have more electrons than that of the Cr_2_TiC_2_O_2_ structure due to the higher unoccupied O orbitals. Therefore, it is possible that just the extra-unoccupied orbitals of O atoms can cause the ferromagnetic to an antiferromagnetic phase transition during the increase of *n*.

### 3.3. Neel Temperature or Curie Temperature of Cr_2_TiC_2_O_n/4_F_2−n/4_

Heisenberg’s model was used to calculate the Neel temperature (T_N_) and Curie temperature (T_C_) of the Cr_2_TiC_2_O_n/4_F_2−n/4_ and Cr_2_TiC_2_O_n/4_ systems. Firstly, the exchange coupling parameters (J_1_, J_2_, and J_3_) were calculated, where J_1_ and J_2_ represent the nearest, next-nearest, and next-next-nearest neighbor exchange coupling parameters, as is shown in Figure 1a. The exchange interaction is calculated by:

$$E_{\mathrm{ex}}=-\underset{i,j}{\sum }J_{1}S_{i}\cdot S_{j}-\underset{i,l}{\sum }J_{2}S_{i}\cdot S_{l}-\underset{i,k}{\sum }J_{3}S_{i}\cdot S_{k}$$

where S_i_ is the magnetic moment of Cr atoms. For the Cr_2_TiC_2_O_n/4_F_2−n/4_ systems, the number of the corresponding couplings for J_1_, J_2_, and J_3_ are 6, 3, and 3, respectively. As shown in Figure 1a, all Cr ions have the same spin directions for FM arrangement. Thus, the total energy of system can be written as Equation (1). For AFM1 arrangement, all the nearest Cr ions have the same spin directions, while Cr ions have the opposite direction with next-nearest and next-next-nearest neighbor Cr ions. For AFM2, the number of same/opposite spin directions for Cr ions with the nearest, next-nearest, and next-next-nearest neighbor Cr ions are 2/4, 2/1, 0/3, respectively. For AFM3, the number of same/opposite spin directions for Cr ions with the nearest, next-nearest, and next-next-nearest neighbor Cr ions are 2/4, 1/2, 3/0 respectively. Thus, the Heisenberg–Hamiltonian equation can be expressed as follows for the FM, AFM1, AFM2, and AFM3 of Cr_2_TiC_2_O_n/4_F_2−n/4_ systems, which is comparable to the previously reported theoretical studies [9,10,34,35]:

E_FM_ = E_0_ − (6J_1_ + 3J_2_ + 3J_3_) S^2^ (1)

E_AFM1_ = E_0_ − (6J_1_ − 3J_2_ − 3J_3_) S^2^ (2)

E_AFM2_ = E_0_ − (−2J_1_ + J_2_ − 3J_3_) S^2^ (3)

E_AFM3_ = E_0_ − (−2J_1_ − J_2_ + 3J_3_) S^2^ (4)

where S is the net magnetic moment of the Cr atom and E_FM_, E_AFM1_, E_AFM2_, E_AFM3_ stand for the total energy of the Cr_2_TiC_2_O_n/4_F_2−n/4_ system with FM, AFM1, AFM2, AFM3 arrangements. E_0_ is the total energy of systems except for exchange energy. From these four equations, J_1_, J_2_, and J_3_ can be calculated as follows:

J_1_ = (E_AFM3_ + E_AFM2_ − E_AFM1_ − E_FM_)/16S^2^ (5)

J_2_ = (E_AFM1_ − E_AFM2_ + E_AFM3_ − E_FM_)/8S^2^ (6)

J_3_ = (E_AFM1_ + 3E_AFM2_ − 3E_AFM3_ − E_FM_)/24S^2^ (7)

The magnetic anisotropy energy (MAE) was calculated by the following equation to investigate the spin directions [36]:

MAE= (E_100_ − E_001_)/N_Cr_

where E_100_ and E_001_ are the total energies of the Cr_2_TiC_2_O_n/4_F_2−n/4_ systems with a spin of the Cr atoms along the 100 and 001 directions. The existence of a positive value for MAE means that the magnetic easy axis of Cr along the c-axis is perpendicular to the layers of the Cr_2_TiC_2_O_n/4_F_2−n/4_ system, while a negative value means that the magnetic easy axis is located in the xy-plane. In addition, N_Cr_ is the number of Cr atoms.

As is presented in Table 1, MAE varies with *n*. For the case when *n* > 4, MAE is negative with a maximum value of −404 μeV at *n* = 0 (Cr_2_TiC_2_O_2_). This result indicates that the magnetic easy axis of Cr belongs to the xy-plane generally, as is shown in Figure 3. For the case when *n* < 4, the extracted MAE value is positive. More specifically, the MAE value of the Cr_2_TiC_2_F_2_ structure is 61 μeV with *n* = 0, which means that the magnetic easy axis of Cr is along the c-axis. For the case when *n* = 4, the configurations of C2 and C3 have a positive value of MAE while the others exhibit a negative value. Such variation of the magnetic easy axis from the c-axis to the xy-plane as *n* increases is considered an important way to tune the magnetic easy axis of Cr_2_TiC_2_O_n/4_F_2−n/4_-based systems.

The exchange coupling parameters (J_1_, J_2_, and J_3_) also vary as *n* is increased, as can be observed in Table 1. These variations of the exchange coupling parameters and MAE could eventually lead to the variation of T_C_ and T_N_. Here, the values of T_C_ and T_N_ for the Cr_2_TiC_2_O_n/4_F_2−n/4_ systems were estimated based on the Heisenberg model by using DFT-derived spin-exchange parameters. Monte Carlo (MC) simulations were also performed by the MCSOLVER package that was developed by Liu and coworkers [37]. The value of T_N_ lies within the range 10~99 K for antiferromagnetic Cr_2_TiC_2_O_n/4_F_2−n/4_ systems with *n* ≤ 4, while the value of T_C_ was calculated within the range 10~47 K for ferromagnetic Cr_2_TiC_2_O_n/4_F_2−n/4_ systems with *n* > 4. In consideration of the random distribution of the surface F and O terminations, such types of configurations may exist in the actual samples. Such a theoretical result is in good agreement with former experimental results concerning the magnetic phase transition temperature of the Cr_2_TiC_2_O_1.3_F_0.8_ structure, which was about 30 K [11,14]. Although both T_N_ and T_C_ for Cr_2_TiC_2_O_n/4_F_2−n/4_ systems are quite lower than the room temperature value, it is quite important to deeply investigate the magnetic properties of Cr-based MXenes.

As far as F atoms are concerned, they can be desorbed under the temperature value of 600 °C. Additionally, both MAE and magnetic phase transition temperature for Cr_2_TiC_2_O_n/4_ systems were calculated in this work, which may also be attained in experimentation [14]. As is shown in Table S1, for the Cr_2_TiC_2_ (*n* = 0) structure, the value of MAE was 27 μeV, which indicates that the magnetic easy axis of Cr is along the c-axis. The value of 443 K was also extracted for T_N_ of Cr_2_TiC_2_, which is considered relatively high for two-dimensional magnetic materials. However, from the previously reported theoretical research, it can be argued that the surface metal atoms on MXenes without termination are active [38]. As a result, H_2_O or O_2_ molecules can be dissociated spontaneously, rendering the stable formation of MXenes without surface terminations impossible.

Since the magnetic easy axis of Cr atoms of both Cr_2_TiC_2_ and Cr_2_TiC_2_O_2_ configurations is along the c-axis, the magnetic easy axis of Cr atoms for all Cr_2_TiC_2_O_n/4_ systems is along the c-axis, as is shown in Table S1, which is different to the Cr_2_TiC_2_O_n/4_F_2−n/4_ systems. A phase transition from AFM to FM states also takes place as *n* is increased from 0 to 8 for Cr_2_TiC_2_O_n/4_ systems. The point of the FM-AFM phase transition is comparable for the cases when *n* = 4 or *n* = 5 for both Cr_2_TiC_2_O_n/4_F_2−n/4_ and Cr_2_TiC_2_O_n/4_ systems. Differently, the acquired values of T_C_ and T_N_ of the Cr_2_TiC_2_O_n/4_ systems are relatively higher than that of the Cr_2_TiC_2_O_n/4_F_2−n/4_ systems, as shown in Figure S5. For *n* ≥ 5, the value of T_C_ for the ferromagnetic Cr_2_TiC_2_O_n/4_ systems varies from 7 to 121 K. Especially for the Cr_2_TiC_2_O_5/4_ (*n* = 5) structure, T_C_ varies from 62~121 K, which is much higher than that of the Cr_2_TiC_2_O_5/4_F_3/4_ configuration (10~47 K). For *n* ≤ 4, the value of T_N_ for the antiferromagnetic Cr_2_TiC_2_O_n/4_ systems lies within the range 9~443 K, which is also higher than that of the Cr_2_TiC_2_O_5/4_F_3/4_ structure in general. Thus, the desorption of F atoms is regarded as a strategic method for increasing the magnetic phase transition temperature of the Cr_2_TiC_2_T_x_ structure.

### 3.4. Impact of n on the Electronic Properties of Cr_2_TiC_2_O_n/4_F_2−n/4_ and Cr_2_TiC_2_O_n/4_ Systems

**Figure 4:**
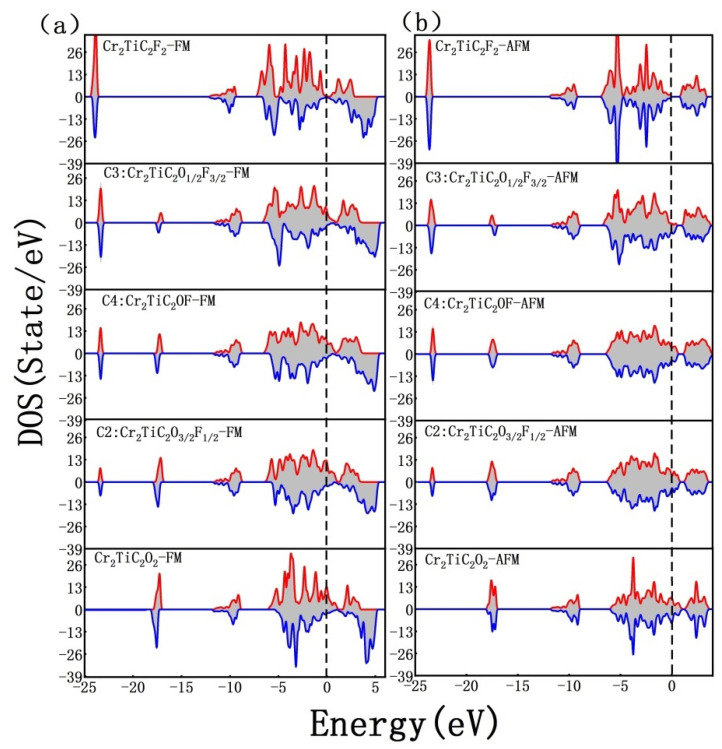
(**a**) Total density of states for FM Cr_2_TiC_2_F_2_, Cr_2_TiC_2_O_1/2_F_3/2_ (C3), Cr_2_TiC_2_OF (C4), Cr_2_TiC_2_O_3/2_F_1/2_ (C2), and Cr_2_TiC_2_O_2_. (**b**) The corresponding total density of states for AFM1 with same structures in panel (a). The configurations with the lowest energy were used here.

The total density of states for both Cr_2_TiC_2_O_n/4_F_2−n/4_ and Cr_2_TiC_2_O_n/4_ systems was calculated for investigating the impact of n on their electronic properties. More specifically, for the Cr_2_TiC_2_F_2_ (*n* = 0) structure, the band gap of its antiferromagnetic arrangement (AFM1) was about 1.1 eV, which is in good agreement with our former results calculated by using the hybrid functional HSE06 method [10]. Interestingly, the ferromagnetic arrangement of the Cr_2_TiC_2_F_2_ (*n* = 0) configuration exhibits a semimetal nature, as is displayed in Figure 4. The application of an external magnetic field can induce the ferromagnetic transition and the acquisition of metallic properties. Thus, the conductivity is anticipated to change dramatically, which renders the Cr_2_TiC_2_F_2_ system a promising material for magnetic sensor applications. The detailed variations of the current–voltage performance with the scale of the Cr_2_TiC_2_F_2_ structure will be analytically examined in our future work. In addition, the T_N_ of the Cr_2_TiC_2_F_2_ structure is quite a lot lower than the room temperature. Attaining such a type of conductor–semiconductor transition induced by magnetization in two-dimensional magnetic materials with higher T_N_ is regarded as a significant assignment in spin electronics.

For the Cr_2_TiC_2_O_2_ (*n* = 8) structure, the extra unfilled orbital of O atoms relative to F atoms leads to a slight Fermi level shift. Thus, both the ferromagnetic and antiferromagnetic arrangements are conductive. However, distinct spin polarization for the ferromagnetic arrangement can be found. As a result, it also may present variations of conductive characteristics with magnetization. As *n* is increased from 0 to 8, an enhanced number of O terminations substitute F ions, and the total density of electronic states is changed. However, such variations of conductive characteristics with magnetization already existed. Thus, from an experimental point of view, despite the fact that the configuration of terminations on the surface of the Cr_2_TiC_2_O_n/4_F_2−n/4_ structure is hard to control and detect, the variations of conductivity with magnetization can be observed. The same pattern also takes place in Cr_2_TiC_2_O_n/4_ systems, as is shown in Figure S6.

## 4. Conclusions

In general, the magnetic properties of the Cr_2_TiC_2_T_x_ structure were simulated by employing the GGA + U approach with the Hubbard U values for Cr and Ti as 4.1 and 3.1 eV, respectively, which were calculated by the linear response method. From the simulated outcomes, it can be argued that the Cr_2_TiC_2_O_n/4_F_2−n/4_ systems with low O content (*n* ≤ 4) are antiferromagnetic, while most of them are ferromagnetic for higher O content systems (*n* ≥ 5). Combined with the random distribution of O atoms, this effect leads to the formation of a spin-glass state for the Cr_2_TiC_2_T_x_ structure, from an experimental point of view. For the Cr_2_TiC_2_O_5/4_F_3/4_ system (*n* = 5), a value of about 2.64 μ_B_ was extracted for M_Cr_, while the Curie temperature was around 10~47 K, which are both in good agreement with experimental results of the Cr_2_TiC_2_O_1.3_F_0.8_ structure. The magnetic moment of Cr atoms is also decreased as *n* becomes bigger. The same trends can be also observed in Cr_2_TiC_2_O_n/4_ systems, after the desorption of F atoms by the Cr_2_TiC_2_O_n/4_F_2−n/4_ structure under the implementation of a relatively high temperature. Additionally, the Neel temperature is decreased as *n* is increased for the Cr_2_TiC_2_O_n/4_ systems generally. For the Cr_2_TiC_2_O_n/4_F_2−n/4_ and Cr_2_TiC_2_O_n/4_ systems, the density of states around the Fermi level changed significantly between ferromagnetic and antiferromagnetic arrangements. This effect indicates that the conductivity can be regulated by a magnetic field. Our results pave the way for tuning the magnetic properties of Cr_2_TiC_2_T_x_-based structures through surface termination techniques and are considered of vital importance to spin electronics applications.

## Figures and Tables

**Table 1:** J_1_, J_2_, J_3_, MAE, T_N_, and T_C_ of Cr_2_TiC_2_O_n/4_F_2−n/4_ systems.

*n*			MAE (μeV)	J_1_ (meV)	J_2_ (meV)	J_3_ (meV)	T_N_/T_C_ (K)
0	C1	AFM1	61	6.61	−0.70	−2.00	49
1	C1	AFM1	68	5.00	−0.31	−0.83	47
2	C1	AFM1	90	2.78	−1.27	0.55	99
	C2	AFM1	51	2.67	−1.27	0.39	92
	C3	AFM1	36	4.15	−0.88	−0.26	77
3	C1	AFM1	51	4.59	−0.55	−0.39	69
	C2	AFM1	1	2.16	−0.76	0.27	62
	C3	AFM1	42	2.33	−1.32	0.66	99
4	C1	AFM1	−5	5.15	−0.71	0.03	92
	C2	AFM1	29	2.51	−0.58	0.02	55
	C3	AFM1	29	2.44	−1.05	0.45	84
	C4	AFM1	−81	1.59	−0.12	−0.15	17
	C5	AFM1	−104	1.35	−0.02	−0.19	10
	C6	AFM1	−28	1.61	−0.45	0.21	40
5	C1	FM	−64	2.83	−0.20	0.24	47
	C2	FM	−4	1.89	0.33	0.07	10
	C3	FM	−3	2.13	−0.01	0.02	25
6	C1	FM	−106	2.25	0.04	0.06	25
	C2	AFM1	−114	2.32	−0.06	−0.11	25
	C3	FM	−165	2.28	0.42	0.09	10
7	C1	FM	−260	3.13	0.37	0.74	40
8	C1	FM	−404	3.76	2.19	−0.49	25

## Data Availability

Restrictions apply to the availability of these data. Data was obtained from Quzhou University and are available from Shaozheng Zhang and Jianhui Yang.

## Supplementary Materials

The following supporting information can be downloaded at: https://www.mdpi.com/article/10.3390/nano12244364/s1, Figure S1: Adsorption sites of Cr_2_TiCO_2_ and total energies of Cr_2_TiC_2_F_2_ and Cr_2_TiC_2_O_2_ with FM and AFM arrangements; Figure S2: Charge of Cr or Ti atoms in Cr_2_TiC_2_O_2_, Cr_2_TiC_2_F_2_, and Cr_2_TiC_2_(OH)_2_ systems under different Hubbard U; Figure S3: The energy differences between AFM1 and FM states (ΔE_AFM1_) of Cr_2_TiC_2_F_2_ and the total magnetic moments of Cr_2_TiC_2_F_2_ FM under different Hubbard U of Cr atoms (UCr); Figure S4: The number of electrons of Cr atoms (e_Cr_), the magnetic moment of Cr atoms (M_Cr_), and energy difference of AFM1, AFM2, AFM3 with FM state of Cr_2_TiC_2_O_n/4_; Figure S5: Variations of T_N_ or T_C_ with n for Cr_2_TiC_2_O_n/4_F_2-n/4_ and Cr_2_TiC_2_O_n/4_; Figure S6: Total density of states for FM and AFM1 Cr_2_TiC_2_, Cr_2_TiC_2_O_1/2_ (C1), Cr_2_TiC_2_O (C5), Cr_2_TiC_2_O_3/2_ (C1); Table S1: J1, J2, J3, MAE, T_N_, and T_C_ of Cr_2_TiC_2_O_n/4_ systems.

## References

[1] Naguib M., Mashtalir O., Carle J., Presser V., Lu J., Hultman L., Gogotsi Y., Barsoum M.W. (2012). Two-Dimensional Transition Metal Carbides. ACS Nano.

[2] Naguib M., Come J., Dyatkin B., Presser V., Taberna P.L., Simon P., Barsoum M.W., Gogotsi Y. (2012). MXene: A promising transition metal carbide anode for lithium-ion batteries. Electrochem. Commun..

[3] Dall’Agnese Y., Lukatskaya M.R., Cook K.M., Taberna P.-L., Gogotsi Y., Simon P. (2014). High capacitance of surface-modified 2D titanium carbide in acidic electrolyte. Electrochem. Commun..

[4] Xiao Y., Zhang W.B. (2020). High throughput screening of M3C2 MXenes for efficient CO2 reduction conversion into hydrocarbon fuels. Nanoscale.

[5] Yang J., Zhang S., Wang A., Wang R., Wang C.-K., Zhang G.-P., Chen L. (2018). High magnetoresistance in ultra-thin two-dimensional Cr-based MXenes. Nanoscale.

[6] Jiang X.T., Kuklin A.V., Baev A., Ge Y.Q., Agren H., Zhang H., Prasad P.N. (2020). Two-dimensional MXenes: From morphological to optical, electric, and magnetic properties and applications. Phys. Rep..

[7] Jing Z., Wang H., Feng X., Xiao B., Ding Y., Wu K., Cheng Y. (2019). Superior Thermoelectric Performance of Ordered Double Transition Metal MXenes: Cr2TiC2T2 (T = -OH or -F). J. Phys. Chem. Lett..

[8] Yang J., Wang A., Zhang S., Wu H., Chen L. (2018). Stability and electronic properties of sulfur terminated two-dimensional early transition metal carbides and nitrides (MXene). Comput. Mater. Sci..

[9] Yang J., Luo X., Zhou X., Zhang S., Liu J., Xie Y., Lv L., Chen L. (2017). Tuning magnetic properties of Cr2M2C3T2 (M = Ti and V) using extensile strain. Computat. Mater. Sci..

[10] Yang J., Zhou X., Luo X., Zhang S., Chen L. (2016). Tunable electronic and magnetic properties of Cr2M’C2T2 (M’ = Ti or V; T=O, OH or F). Appl. Phys. Lett..

[11] Hantanasirisakul K., Anasori B., Nemsak S., Hart J.L., Wu J., Yang Y., Chopdekar R.V., Shafer P., May A.F., Moon E.J. (2020). Evidence of a magnetic transition in atomically thin Cr2TiC2Tx MXene. Nanoscale Horiz..

[12] Frey N.C., Kumar H., Anasori B., Gogotsi Y., Shenoy V.B. (2018). Tuning Noncollinear Spin Structure and Anisotropy in Ferromagnetic Nitride MXenes. ACS Nano.

[13] Kumar H., Frey N.C., Dong L., Anasori B., Gogotsi Y., Shenoy V.B. (2017). Tunable Magnetism and Transport Properties in Nitride MXenes. ACS Nano.

[14] Hart J.L., Hantanasirisakul K., Lang A.C., Li Y.Y., Mehmood F., Pachter R., Frenkel A.I., Gogotsi Y., Taheri M.L. (2021). Multimodal Spectroscopic Study of Surface Termination Evolution in Cr2TiC2Tx MXene. Adv. Mater. Interfaces.

[15] Yang J., Zhang S., Li L., Wang A., Zhong Z., Chen L. (2019). Rationally designed high-performance spin-filter based on two-dimensional half-metal Cr2NO2. Matter.

[16] Si C., Zhou J., Sun Z.M. (2015). Half-Metallic Ferromagnetism and Surface Functionalization-Induced Metal-Insulator Transition in Graphene-like Two-Dimensional Cr2C Crystals. Acs Appl. Mater. Interfaces.

[17] Sun Q., Fu Z.M., Yang Z.X. (2020). Tunable magnetic and electronic properties of the Cr-based MXene (Cr2C) with functional groups and doping. J. Mag. Mag. Mater..

[18] Ma X.F., Mi W.B. (2020). Surface Functionalization Tailored Electronic Structure and Magnetic Properties of Two-Dimensional CrC2 Monolayers. J. Phys. Chem. C.

[19] He J.J., Ding G.Q., Zhong C.Y., Li S., Li D.F., Zhang G. (2019). Cr2TiC2-based double MXenes: Novel 2D bipolar antiferromagnetic semiconductor with gate-controllable spin orientation toward antiferromagnetic spintronics. Nanoscale.

[20] Frey N.C., Bandyopadhyay A., Kumar H., Anasori B., Gogotsi Y., Shenoy V.B. (2019). Surface-Engineered MXenes: Electric Field Control of Magnetism and Enhanced Magnetic Anisotropy. ACS Nano.

[21] He J.J., Lyu P.B., Sun L.Z., Garcia A.M., Nachtigall P. (2016). High temperature spin-polarized semiconductivity with zero magnetization in two-dimensional Janus MXenes. J. Mater. Chem. C.

[22] Serrano G., Poggini L., Briganti M., Sorrentino A.L., Cucinotta G., Malavolti L., Cortigiani B., Otero E., Sainctavit P., Loth S. (2020). Quantum dynamics of a single molecule magnet on superconducting Pb(111). Nat. Mater..

[23] Je M., Lee Y., Chung Y.C. (2016). Structural stability and electronic properties of multi-functionalized two-dimensional chromium carbides. Thin Solid Films.

[24] Kresse G., Joubert D. (1999). From ultrasoft pseudopotentials to the projector augmented-wave method. Phys. Rev. B.

[25] Kresse G., Hafner J. (1994). Ab-initio molecular-dynamics simulation of the liquid-metal amorphous semiconductor transition in germanium. Phys. Rev. B.

[26] Kresse G., Furthmuller J. (1996). Efficient iterative schemes for ab initio total-energy calculations using a plane-wave basis set. Phys. Rev. B.

[27] Kresse G., Furthmuller J. (1996). Efficiency of ab-initio total energy calculations for metals and semiconductors using a plane-wave basis set. Comput. Mater. Sci..

[28] Monkhorst H.J., Pack J.D. (1976). Special points for brillouin-zone integrations. Phys. Rev. B.

[29] Perdew J.P., Burke K., Ernzerhof M. (1997). Generalized gradient approximation made simple. Phys. Rev. Lett..

[30] Anisimov V.I., Aryasetiawan F., Lichtenstein A.I. (1997). First-principles calculations of the electronic structure and spectra of strongly correlated systems: The LDA C U method. J. Phys. Condens. Matter.

[31] Cococcioni M., de Gironcoli S. (2005). Linear response approach to the calculation of the effective interaction parameters in the LDA+U method. Phys. Rev. B.

[32] Zhang Y.G., Cui Z., Sa B.S., Miao N.H., Zhou J., Sun Z.M. (2022). Computational design of double transition metal MXenes with intrinsic magnetic properties. Nanoscale Horiz..

[33] Anderson P.W. (1950). Antiferromagnetism. Theory of superexchange interaction. Phys. Rev..

[34] Li S., He J.J., Grajciar L., Nachtigall P. (2021). Intrinsic valley polarization in 2D magnetic MXenes: Surface engineering induced spin-valley coupling. J. Mater. Chem. C.

[35] Caneschi A., Gatteschi D., Totti F. (2015). Molecular magnets and surfaces: A promising marriage. A DFT insight. Coord. Chem. Rev..

[36] Zhuang H.L., Kent P.R.C., Hennig R.G. (2016). Strong anisotropy and magnetostriction in the two-dimensional Stoner ferromagnetFe3GeTe2. Phys. Rev. B.

[37] Liu L., Ren X., Xie J.H., Cheng B., Liu W.K., An T.Y., Qin H.W., Hu J.F. (2019). Magnetic switches via electric field in BN nanoribbons. Appl. Surf. Sci..

[38] Yang J., Zhang S., Ji J., Wei S. (2015). Adsorption Activities of O, OH, F and Au on Two-Dimensional Ti2C and Ti3C2 Surfaces. Acta Phys.Chim. Sin..

