# Supplementary material for: Tuning the Magnetic Properties of Cr2TiC2Tx through Surface Terminations: A Theoretical Study
Source: Nanomaterials (Basel). 2022 Dec 7;12(24):4364. doi: 10.3390/nano12244364 (PMC9781736; doi:10.3390/nano12244364)
Supplement: Supplementary file 1 [file nanomaterials-12-04364-s001.zip › nanomaterials-1985199-supplementary.pdf]

*nanomaterials*

MDPI

*Support information*

# Tuning the Magnetic Properties of $Cr_2TiC_2T_x$ Through Surface Terminations: A Theoretical Study

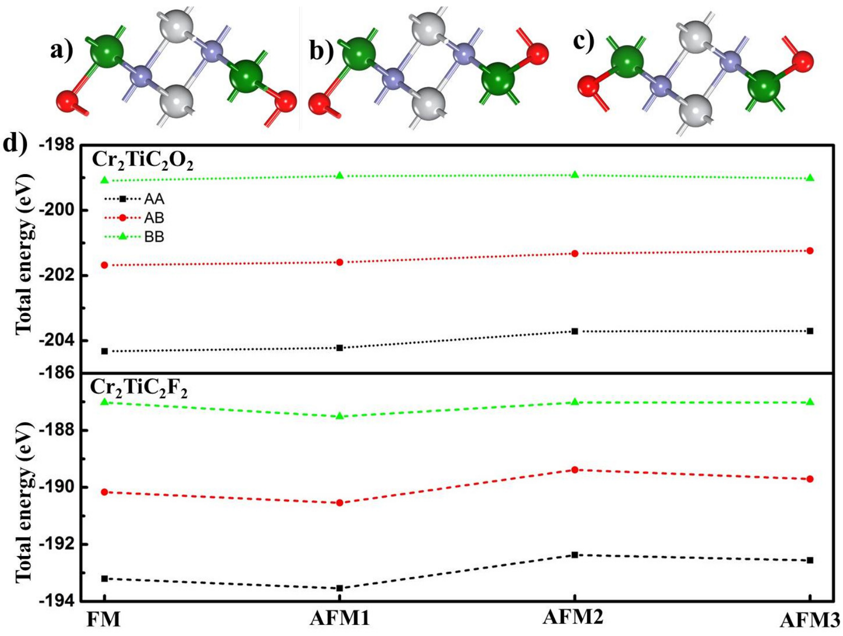


**Figure S1.** Adsorption sites of $Cr_2TiCO_2$. a) Two O atoms on hollow site A under which there is a Ti atom (model AA), b) One O atom on hollow site A and one O atom on hollow site B under which there is a C atom (model AB), c) Two O atoms on hollow site B (model AB). d) Total energies of $Cr_2TiC_2F_2$ and $Cr_2TiC_2O_2$ with FM and AFM arrangements.

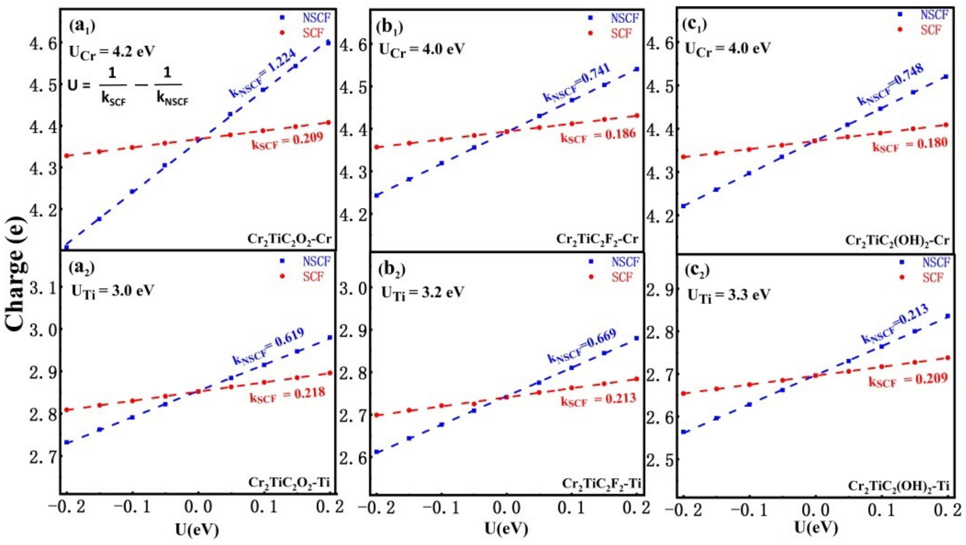


**Figure S2.** Charge of Cr or Ti atoms in $Cr_2TiC_2O_2$, $Cr_2TiC_2F_2$, and $Cr_2TiC_2(OH)_2$ systems under different Hubbard U.

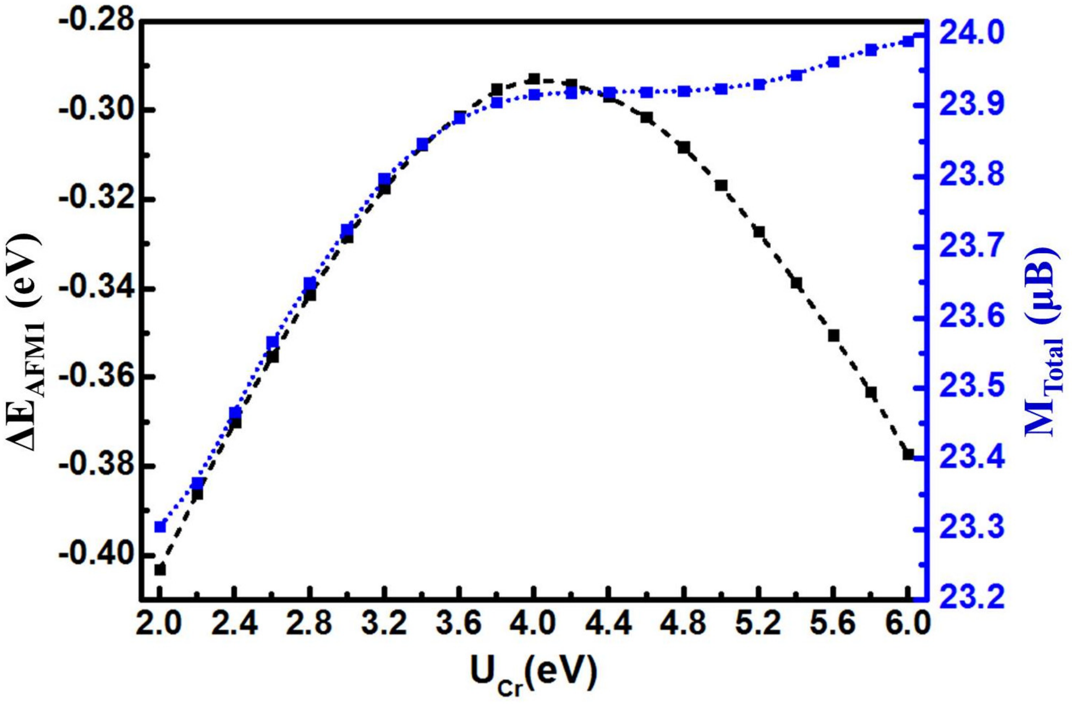


**Figure S3.** The energy differences between AFM1 and FM states ($\Delta E_{AFM1}$) of $Cr_2TiC_2F_2$ and the total magnetic moments of $Cr_2TiC_2F_2$ FM under different Hubbard U of Cr atoms ($U_{Cr}$).

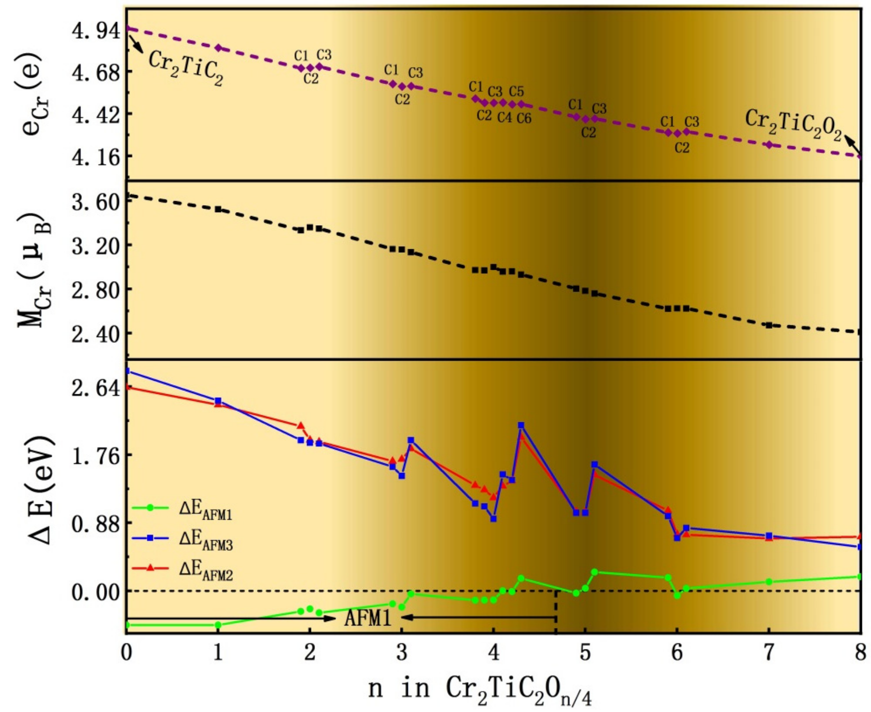


**Figure S4.** (Top panel) The number of electrons of Cr atoms ($e_{Cr}$). (Middle panel) the magnetic moment of Cr atoms ($M_{Cr}$). (Down panel)Energy difference of AFM1, AFM2, AFM3 with FM state of $Cr_2TiC_2O_{n/4}$.

**Table S1.** $J_1$, $J_2$, $J_3$, MAE, $T_N$, and $T_C$ of $Cr_2TiC_2O_{n/4}$ systems.

| | | | MAE(μeV) | $J_1$(meV) | $J_2$(meV) | $J_3$(meV) | $T_N$/$T_C$(K) |
|---|---|---|---|---|---|---|---|
| 0 | C1 | AFM1 | 27 | 20.56 | -4.54 | 0.45 | 443 |
| 1 | C1 | AFM1 | 87 | 17.94 | -2.45 | -0.32 | 311 |
| 2 | C1 | AFM1 | 79 | 14.30 | -1.80 | -0.82 | 233 |
| | C2 | AFM1 | 68 | 14.24 | -1.37 | -0.79 | 211 |
| | C3 | AFM1 | 48 | 15.09 | -0.60 | -1.87 | 151 |
| 3 | C1 | AFM1 | 44 | 13.28 | -1.04 | 0.68 | 225 |
| | C2 | AFM1 | 64 | 11.81 | 0.04 | -1.98 | 69 |
| | C3 | AFM1 | 24 | 11.99 | -0.66 | -0.90 | 144 |
| 4 | C1 | AFM1 | 55 | 13.78 | 0.04 | 1.46 | 203 |
| | C2 | AFM1 | 14 | 9.94 | -0.15 | 0.07 | 122 |
| | C3 | AFM1 | 5 | 9.94 | -1.03 | 1.05 | 188 |
| | C4 | AFM1 | 34 | 7.83 | 1.09 | -2.17 | 32 |
| | C5 | AFM1 | 39 | 8.75 | 0.66 | -1.77 | 9 |
| | C6 | AFM1 | 9 | 9.10 | 0.78 | -1.90 | 12 |
| 5 | C1 | FM | 39 | 10.04 | 0.77 | 1.49 | 121 |
| | C2 | FM | 40 | 6.95 | 0.36 | -0.04 | 62 |
| | C3 | FM | 13 | 7.17 | -0.08 | -0.19 | 84 |
| 6 | C1 | FM | 19 | 5.24 | -0.40 | 0.69 | 99 |
| | C2 | AFM1 | 33 | 5.13 | -0.07 | -0.49 | 47 |
| | C3 | FM | 135 | 6.38 | 1.67 | -0.09 | 7 |
| 7 | C1 | FM | 186 | 4.43 | 0.56 | 0.53 | 17 |

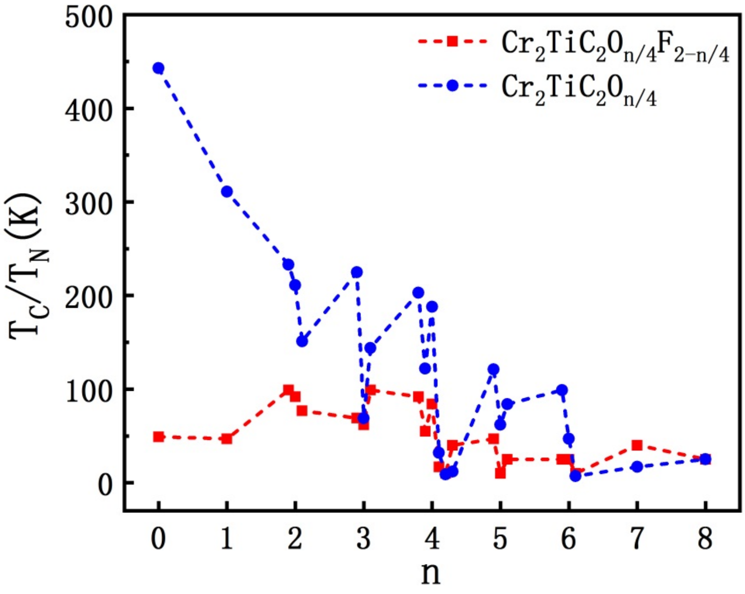


**Figure S5.** Variations of $T_N$ or $T_C$ with n for $Cr_2TiC_2O_{n/4}F_{2-n/4}$ and $Cr_2TiC_2O_{n/4}$.

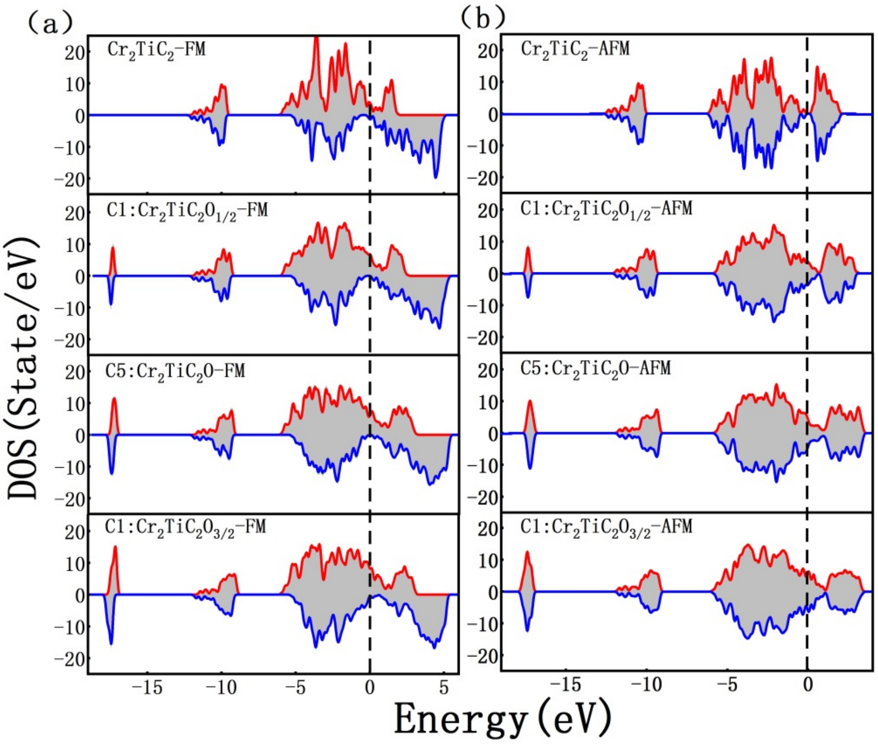


**Figure S6.** Total density of states for FM and AFM1 $Cr_2TiC_2$, $Cr_2TiC_2O_{1/2}$ (C1), $Cr_2TiC_2O$ (C5), $Cr_2TiC_2O_{3/2}$ (C1). The configurations with the lowest energy were used here.
